# JAK2-Centered Interactome Hotspot Identified by an Integrative Network Algorithm in Acute Stanford Type A Aortic Dissection

**DOI:** 10.1371/journal.pone.0089406

**Published:** 2014-02-24

**Authors:** Sun Pan, Duojiao Wu, Andrew E. Teschendorff, Tao Hong, Linyan Wang, Mengjia Qian, Chunsheng Wang, Xiangdong Wang

**Affiliations:** 1 Department of Cardiac Surgery, Zhongshan Hospital, Fudan University, Shanghai, China; 2 Biomedical Research Center, Zhongshan Hospital, Fudan University, Shanghai, China; 3 UCL Cancer Institute, University College London, London, United Kingdom; 4 CAS-MPG Partner Institute for Computational Biology, Shanghai, China; University of Jaén, Spain

## Abstract

The precise mechanisms underlying dissections, especially those without connective tissue diseases or congenital vascular diseases, are incompletely understood. This study attempted to identify both the expression profile of the dissected ascending aorta and the interactome hotspots associated with the disease, using microarray technology and gene regulatory network analysis. There were 2,737 genes differentially expressed between patients with acute Stanford type A aortic dissection and controls. Eight interactome hotspots significantly associated with aortic dissection were identified by an integrative network algorithm. In particular, we identified a JAK2-centered expression module, which was validated in an independent gene expression microarray data set, and which was characterized by over-expressed cytokines and receptors in acute aortic dissection cases, indicating that JAK2 may play a key role in the inflammatory process, which potentially contributes to the occurrence of acute aortic dissection. Overall, the analytical strategy used in this study offered the possibility to identify functional relevant network modules and subsequently facilitated the biological interpretation in the complicated disease.

## Introduction

Aortic dissection is a life threatening disease, characterized by redirection of blood flow from the aorta through an intimal tear into the media of the aortic wall, which at the end results in two blood stream channels, the true and false lumen. It is classified anatomically as Stanford type A if the ascending aorta is involved. Patients are considered to have an acute aortic dissection (AAD) when the process is less than 14 days. Aortic dissection is the most frequently diagnosed lethal condition of the aorta. The mortality before admission was about 20%, and a total of 68% of the hospitalized patients died within 48 h of admission [1].

It is proposed that aortic dissection is the end process of an array of different pathological processes, many of which promote weakening of or increased stress on, the aortic wall, or both. The sequence of events might begin with a tear in a damaged intima [2]. In spite of the literatures on aortic dissection, the precise mechanisms underlying dissections, especially those without connective tissue diseases or congenital vascular diseases, are incompletely understood. Hypotheses include structural weakening of extracellular matrix, changes in transforming growth factor-beta (TGF beta) signaling, dysfunction in vascular smooth muscle cells as well as chronic inflammation. However, the emergency nature of the disease does not easily lend it to study. Still little is known about the underlying defects.

To investigate the molecular profile at the site of dissected ascending aorta, we used microarray based genome-wide expression profiling. Subsequent application of supervised statistical methods, enables gene-by-gene comparison of differential expression. However, human disease states are increasingly considered to be caused not by singular biochemical alterations but instead result from the multifactorial regulation of gene expression acting in biological systems [3].

Network-based methods provide powerful alternatives of systematic analysis of complex diseases and identification of dysfunctional modules and candidate disease genes. The availability of genome-wide data of high-throughput experiments provides us with new opportunity to explore the hypothesis by analyzing the disease-related biomolecular networks, which are expected to bridge genotypes and disease phenotypes and further reveal the biological mechanisms of complex diseases [4]. To aid the biological interpretation of the dataset resulting from microarray experiment, we used system biology approaches including a novel integrative network algorithm to analyze the differential expression at the level of an interaction network in the aortic tissue in Stanford type A AAD.

## Materials and Methods

### Sample Collection

#### Ethics statement

All studies were conducted under protocols approved by the Ethics Committee of Zhongshan Hospital, Fudan University (Shanghai, China). Written informed consent was obtained from the patients or their relatives.

#### Patients and specimens

Seven consecutive patients (6 males and 1 female, mean age 42.0±17.0 years) underwent emergency surgery for Stanford type A AAD were recruited. Acute aortic dissection is defined as dissection detected within 2 weeks of the onset of symptoms. Patients with Marfan syndrome, Ehlers-Danlos syndrome, and other known connective tissue disorders were excluded. None of the patients had bicuspid aortic valve, aortic coarctation, Takayasu’s arteritis, coronary artery disease or cocaine use. Dissected ascending aorta specimens were obtained through operation, during which the tear site was identified and a strip of aortic wall including the tear site was carefully excised. Any adherent mural thrombus was removed from the aortic wall. Each sample was divided into two parts. One part was used for histological assessment with HE stain, and the other was used for RNA isolation. The tissue specimens used for RNA isolation were free of macroscopic thrombus or blood and were snap-frozen in liquid nitrogen upon collection. As control samples, normal ascending aorta specimens were collected from 5 male organ donors (mean age 38.6±4.8 years). Normal control samples were treated in the exactly same manner as the test samples. All patients and controls were of Asian origin. Detailed clinical information of the individuals enrolled in the study is shown in table 1. There are no statistically significant differences of the demographics between two groups.

**Table 1:** Demographic and clinical characteristics of patients and controls included in this study.

Parameter	patients(n = 7)	controls(n = 5)
Male/female	6/1	5/0
Age		
Mean age, y (SD)	42.0(17.0)	38.6(4.8)
Median age, y(Range)	40(18–67)	39(33–45)
Classification of Blood Pressure* n (%)		
Normal	1(14)	3(60)
Prehypertension	2(29)	2(40)
Hypertension, Stage 1	2(29)	0(0)
Hypertension, Stage 2	2(29)	0(0)
Smoking, n (%)	2(29)	2(40)
Pregnancy, n (%)	1(14)	0(0)
Diabetes, n (%)	1(14)	0(0)
Connective tissue disorders, n (%)	0(0)	0(0)
Hereditary vascular disease, n (%)	0(0)	0(0)
Takayasu’s arteritis, n (%)	0(0)	0(0)
Cocaine use, n (%)	0(0)	0(0)
Deceleration trauma, n (%)	0(0)	0(0)
Iatrogenic manoeuvre, n (%)	0(0)	0(0)
Coronary artery disease, n (%)	0(0)	0(0)
Chronic obstructive disease, n (%)	0(0)	0(0)

*Criteria of hypertension according to JNC 7 blood pressure category. The range of systolic blood pressure (SBP) and diastolic blood pressure (DBP) values for the different stages was as follows:

Normal, SBP<120 and DBP<80 mmHg.

Prehypertension, SBP 120–139 or DBP 80–89 mmHg.

Stage 1 Hypertension, SBP 140–159 or DBP 90–99 mmHg.

Stage 2 Hypertension, SBP≥160 or DBP≥100 mmHg.

#### Total RNA isolation

Aorta tissue specimens were pulverized under liquid nitrogen, and total RNA was extracted from grinned tissue samples with TRIzol reagent (Invitrogen, Carlsbad, USA). Subsequent washing and elution steps were performed according to the manufacturer’s protocol. Total RNA was quantified with NanoDrop2000 spectrophotometer (Thermo Scientific, Wilmington, USA) and the quality and integrity was confirmed by electrophoresis of 0.5 µg isolated RNA on 1% formamide agarose gels.

#### Microarray experiment

Total RNA was amplified into cRNA and biotinylated by in vitro transcription using the Illumina TotalPrep RNA Amplification Kit (Ambion, Applied Biosystems, Foster City, CA, USA). Biotinylated cRNAs were purified, fragmented, and subsequently hybridized to a HumanHT-12 v4 Expression BeadChip (Illumina, San Diego, CA, USA). After scanning of hybridized BeadChip, quantitation of slide images were performed using Illumina’s BeadArray software and the raw data were output. All experimental processes were carried out according to the manufacturer’s protocol.

### Data Analysis

**Figure 1:**
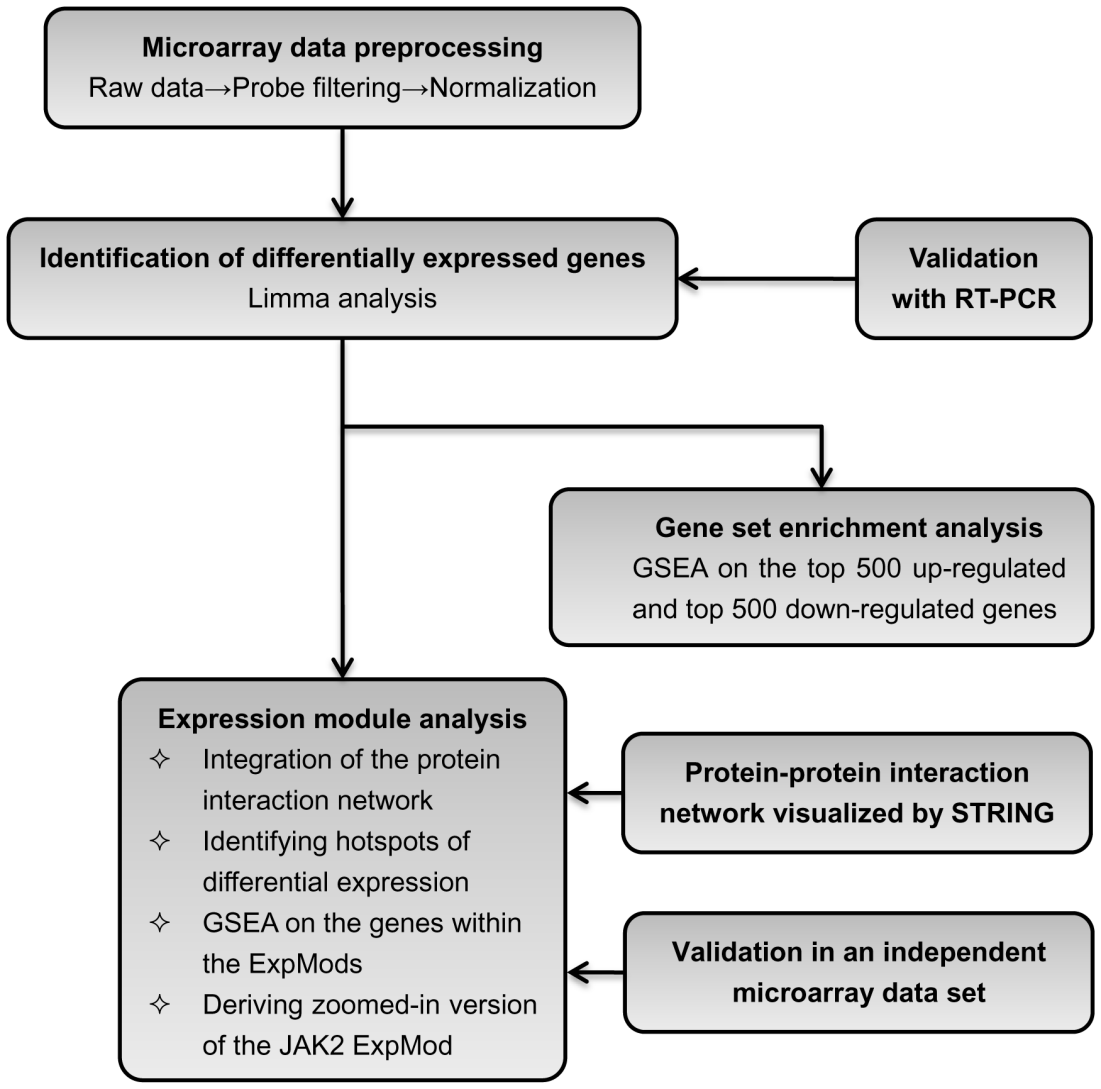
Experimental design. The flowchart schematizes the experimental steps of the statistical analysis of microarray data.

All of raw data have been deposited in NCBI’s Gene Expression Omnibus and are accessible through GEO Series accession number GSE52093 (http://www.ncbi.nlm.nih.gov/geo/query/acc.cgi?acc=GSE52093). The overall data analysis process involved is summarized in figure 1.

#### Quality control and normalization

Background corrected data was filtered for probes with P-values of detection <0.05 in at least 5 of the 12 samples. Data was then quantile normalized and values less than 0 were replaced with the minimum non-zero positive value, so that subsequent log2 transformation would not introduce NA’s. Only probes that were annotated to an Entrez gene ID were kept for further analysis. Expression profiles of probes mapping to the same Entrez gene ID were averaged.

#### Differential gene expression analysis

To find differentially expressed genes (DEGs) between the 7 cases and 5 controls, we used regularized t-statistics derived from within an empirical Bayesian framework, as implemented in the Limma Bioconductor package [5]. Associated P-values were then corrected for multiple testing using estimates for the False Discovery Rate (FDR) using the q-value method [6].

#### Validation of expression

The expression of selected genes was quantified in real time PCR to validate the microarray data. Aliquots of the same total RNA as for microarray experiment were converted into cDNA by reverse transcription. Real-time PCR reactions were performed in 96-well plates with a real-time PCR system (Mastercycler ep realplex, Eppendorf, Hamburg, Germany) and a SYBR Premix Ex Taq™ Kit (TaKaRa, Tokyo, Japan) according to the manufacturer’s protocol. Quantitative analysis was performed using the comparative CT (2^−ΔΔCT^) method [7], [8].

#### Gene set enrichment analysis and expression modules analysis

Gene set enrichment analysis (GSEA) was performed on the top 500 up-regulated (in cases) and top 500 down-regulated (in cases) genes from the Limma analysis using the Molecular Signatures Database (www.broadinstitute.org/gsea/msigdb) [9]. In addition to simple GSEA, we performed a functional supervised analysis, called Hotspots/Expression Modules analysis, whereby feature selection is performed at the level of subnetworks within a human interactome. Specifically, we investigated if the interactome is characterized by hotspots of differential expression, defined as subnetworks where a relatively large number of genes are differentially expressed. As an interactome, we used the protein interaction network from PATHWAY COMMONS (www.pathwaycommons.org), which includes the Human Protein Reference Database (HPRD) interaction network, the Cell Map, the NCI Nature Interaction Database, the INTACT (“interactome”) and MINT (“The Molecular Interactions Network database”) (www.pathwaycommons.org) [10]. Integration of the interactome with the genes present on the array resulted in a network consisting of 8434 proteins with over 300,000 non-redundant documented interactions.

To identify hotspots of differential expression, we assigned edge weights to the network according to the combined strength of the limma t-statistics of the two nodes making up the edge, i.e.
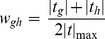
Where *t_g_* labels the limma t-statistic of gene *g* (derived using limma and using case/control status as our phenotype). Thus, *w_gh_* represents the weight of the edge between genes *g* and *h* in the underlying protein interaction network (*PIN*), and 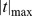 is a normalization factor given by the maximum absolute t-statistic assigned by the test to any gene in the network. Note that if *g* and *h* do not interact we set *w_gh_* = 0. This yields a weighting of each edge between 0 and 1, with larger weights denoting two neighbors both significantly associated with the phenotype of interest. With the choice of edge weighting regime above, the next task is to search for submodules of exceptionally large average weight density, given their topological structure. This average weight density we call modularity [11].

Because of the natural way in which proteins often lie in many distinct functional pathways, we chose not to apply a module detection algorithm that yields mutually exclusive modules. Moreover, inference of modules in large-scale networks can be computationally demanding and unstable. Thus, we adopted a greedy approach in the spirit of other previous works [12], which have shown that a greedy approach is scalable while also successfully identifying biologically relevant modules. Briefly, the greedy approach searches for modules starting out from seed nodes, defined as the nodes with the largest statistics or alternatively those with the largest average weights (i.e the average over nearest neighbors). Both rankings are possible, although the former leads to less redundancy in the sense that seeds are more spread out across the network. The top 100 genes ranked by absolute statistics were declared as seeds (these all passed an FDR correction threshold of 0.05). To identify modules around these seeds we used the spin-glass algorithm [13], which allows community detection in weighted networks specified by a weighted adjacency matrix *W*. The algorithm proceeds using a simulated annealing procedure as implemented in the *spinglass.community* function of the *igraph* R package. Default paramaters were used throughout except for γ, which was set to be γ = 0.5, since this choice leads modules of optimal size as described and evaluated in [14].

Although the modules/communities inferred by the spin-glass algorithm represent subnetworks with modularity values larger than the network average, it is important to further establish the statistical significance of the modularity values associated with the inferred communities. Since the modularity *M* of each community *C* is simply the total weight of all edges in *C*, one can assess the statistical significance by permuting the node statistics (i.e. the absolute Limma t-statistics) over the network and recomputing the modularity values for each of the original communities. We performed 1000 permutations to obtain an adjusted *P*-value for each separate module. Those modules which are significant at the 5% level, we refer to as Hotspots/Expression Modules, or simply as ExpMods. On the genes within the ExpMods, GSEA was further performed to identify enriched gene lists. Among the top ranked ExpMods, zoomed-in version of the JAK2 ExpMod was derived.

#### Protein-protein interaction visualized by STRING

Search Tool for the Retrieval of Interacting Genes (STRING, http://string-db.org/), which is a database of known and predicted protein interactions including direct (physical) and indirect (functional) associations, is used to picture an additional protein-protein interaction involving JAK2. Totally 112 genes including JAK2, which were identified through Expression Modules Analysis, were submitted into STRING online software to visualize the protein-protein interaction.

#### Validation of the JAK2 expression module

The JAK2 expression module was validated in an independent gene expression data set, including 8 samples from 4 patients with AAD and 4 controls [15].

## Results

### Histological Assessment Using HE Stain

The aortas of the patients with AAD showed medial degeneration affecting primarily the elastic fibers associated with focal accumulation of basophilic ground substance (figure S1A). In one patient, there was band of smooth muscle cell loss in the aortic media (figure S1B). HE staining did not display demonstrable presence of inflammatory cells with typical appearance in the aortic media of patients with AAD.

### Differential Gene Expression between Aortic Cases and Controls

Raw data was subjected to quality control procedures, including intra and inter-array normalization, as described in Material and Methods. We identified a total of 22,043 probes that were expressed at significant levels in at least 5 of the 12 samples, constituting 46.6% (22043/47323) of all probes on the array. Probe level data was summarized at the gene-level using Entrez gene annotations, and differentially expressed genes (DEGs) were subsequently identified using an empirical Bayes method that obtains regularized t-statistics for each gene (Materials and Methods). We observed a strong association between gene expression and case control status, as illustrated by the histogram of *P*-values from the Bayesian regularized t-test analysis, with 2,737 Entrez gene Ids significant at a False Discovery Rate (FDR) <0.05 (figure S2). Gene Set Enrichment Analysis using the hypergeometric test on the top 500 ranked upregulated genes (all FDR <0.05) revealed enrichment of cell-cycle genes (table S1), whereas on the top 500 ranked downregulated genes (all FDR <0.05), we observed enrichment of terms relevant to the biology of aortic disease, e.g. unstable atherosclerotic plaque [16] and vascular smooth muscle contraction (table S2).

Summarized expression data and statistics for the top 25 genes are shown in table 2, in order of decreasing significance. Among the 25 genes, CDC45L, ST14, CKAP2L, TIMP1, PHLDA1, LHFPL2, PCSK1, GINS2, TMEM158, TSPANS, ECT2, CCDC34, VARS, CDC7, NT5DC2, LOC646993 and PPIF are up-regulated; while RYR2, C5orf24, JAK2, REEP1, THSD4, BRP44L, RNF115 and BAMBI are down-regulated. Some of the differentially expressed genes are very interesting. For instance, CKAP2L gene encodes cytoskeleton associated with protein 2-like protein. The protein encoded by TIMP1 gene is the natural inhibitor of matrix metalloproteinases (MMPs). The protein encoded by THSD4 gene promotes FBN1 matrix assembly and attenuates TGF beta signaling. BAMBI gene encodes a transmembrane glycoprotein related to the type I receptors of the TGF beta family. JAK2 gene encodes a non-receptor tyrosine kinase, a member of the Janus kinase family, which is important in phosphorylation of cytokine receptors.

**Table 2:** The top 25 most significant genes from Limma analysis.

EntrezID	Symbol	AvExp^a^(Control)	AvExp(Case)	logFC^b^	t^c^	P-value	q-value
8318	CDC45L	−10.60497055	6.888526437	17.4935	29.62106	3.38E-12	2.44E-08
6768	ST14	−10.60497055	4.485981521	15.09095	20.89494	1.73E-10	6.25E-07
150468	CKAP2L	−10.60497055	5.637801523	16.24277	19.80968	3.14E-10	7.56E-07
7076	TIMP1	13.0689603	14.25878751	1.189827	10.94989	2.02E-07	0.000318
22822	PHLDA1	5.587196746	8.027978339	2.440782	10.86172	2.20E-07	0.000318
10184	LHFPL2	7.917215388	9.290336816	1.373121	10.38349	3.53E-07	0.000426
6262	RYR2	7.631018078	5.17677854	−2.45424	−9.46391	9.23E-07	0.000927
5122	PCSK1	−5.768921911	4.842599755	10.61152	9.366506	1.03E-06	0.000927
51659	GINS2	2.717290548	7.332656805	4.615366	9.13514	1.33E-06	0.001042
25907	TMEM158	6.047223743	9.527182627	3.479959	9.059868	1.44E-06	0.001042
134553	C5orf24	8.186755599	6.533418616	−1.65334	−8.56449	2.54E-06	0.001672
3717	JAK2	8.57895724	6.802215907	−1.77674	−8.35271	3.27E-06	0.001969
65055	REEP1	9.588972723	5.917460093	−3.67151	−8.07974	4.54E-06	0.002356
10098	TSPAN5	6.806365429	8.258852748	1.452487	8.076383	4.56E-06	0.002356
79875	THSD4	5.94390384	4.081214951	−1.86269	−7.91683	5.56E-06	0.002555
1894	ECT2	4.61023265	6.977634015	2.367401	7.902448	5.66E-06	0.002555
91057	CCDC34	6.09221058	8.336252794	2.244042	7.849065	6.04E-06	0.00257
51660	BRP44L	10.0463896	8.971708734	−1.07468	−7.62843	7.98E-06	0.003019
7407	VARS	5.131741292	6.826224311	1.694483	7.576624	8.53E-06	0.003019
27246	RNF115	10.0887354	8.477502335	−1.61123	−7.56481	8.66E-06	0.003019
8317	CDC7	4.496631066	6.438868807	1.942238	7.550876	8.82E-06	0.003019
64943	NT5DC2	6.258132602	8.79440855	2.536276	7.507439	9.32E-06	0.003019
646993	LOC646993	2.044714874	5.264715435	3.220001	7.484235	9.61E-06	0.003019
25805	BAMBI	10.45604266	8.230870464	−2.22517	−7.41575	1.05E-05	0.003161
10105	PPIF	−8.4927706	3.706250953	12.19902	7.360952	1.13E-05	0.003233

a AvExp: average expression.

b logFC: log2-fold-change (i.e a logFC>1 means fold-change larger than 2).

c t: regularized Limma t-statistic.

**Figure 2:**
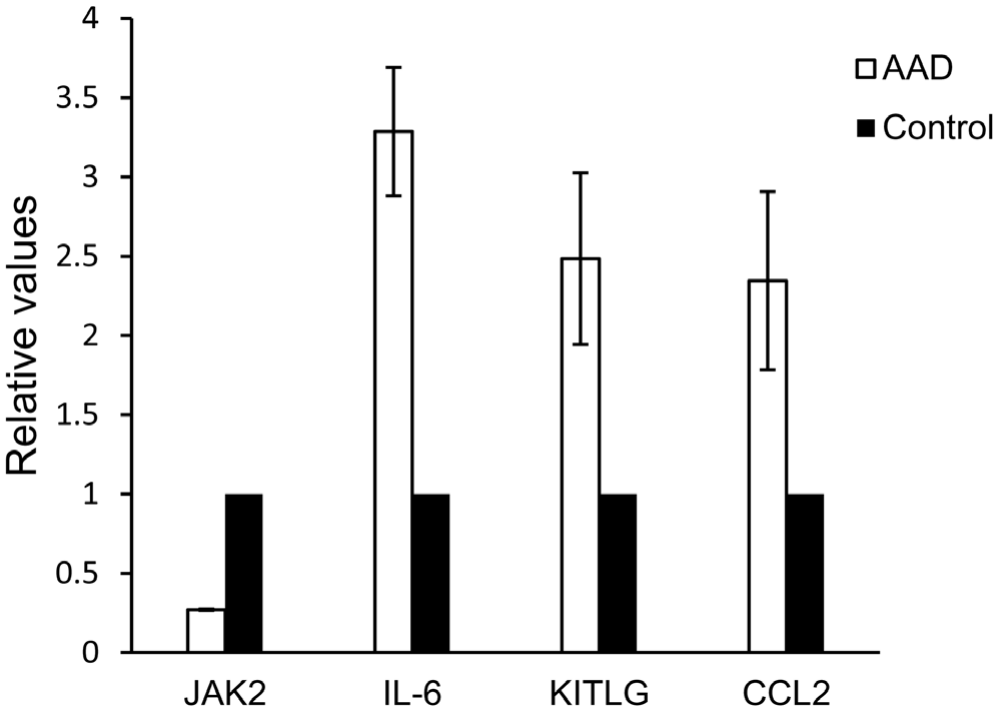
Validation of gene expression by real-time PCR. The expression of JAK2, IL-6, KITLG and CCL2 in the dissected aorta was validated by real-time PCR analyses. Expression levels in control aortas were set to 1. Data represent the mean ± SEM.

Real-time quantitative PCR using aliquots of the same total RNA as for microarray analysis confirmed differential expression of selected genes from the gene list (e.g. JAK2, IL-6, KITLG and CCL2) in AAD patients (figure 2). Confirming the microarray data, we found that JAK2 was down-regulated, while IL-6, KITLG and CCL2 were up-regulated in dissected samples. The results demonstrate agreement between microarray and RT-PCR data.

### Interactome Hotspots/Expression Modules

**Figure 3:**
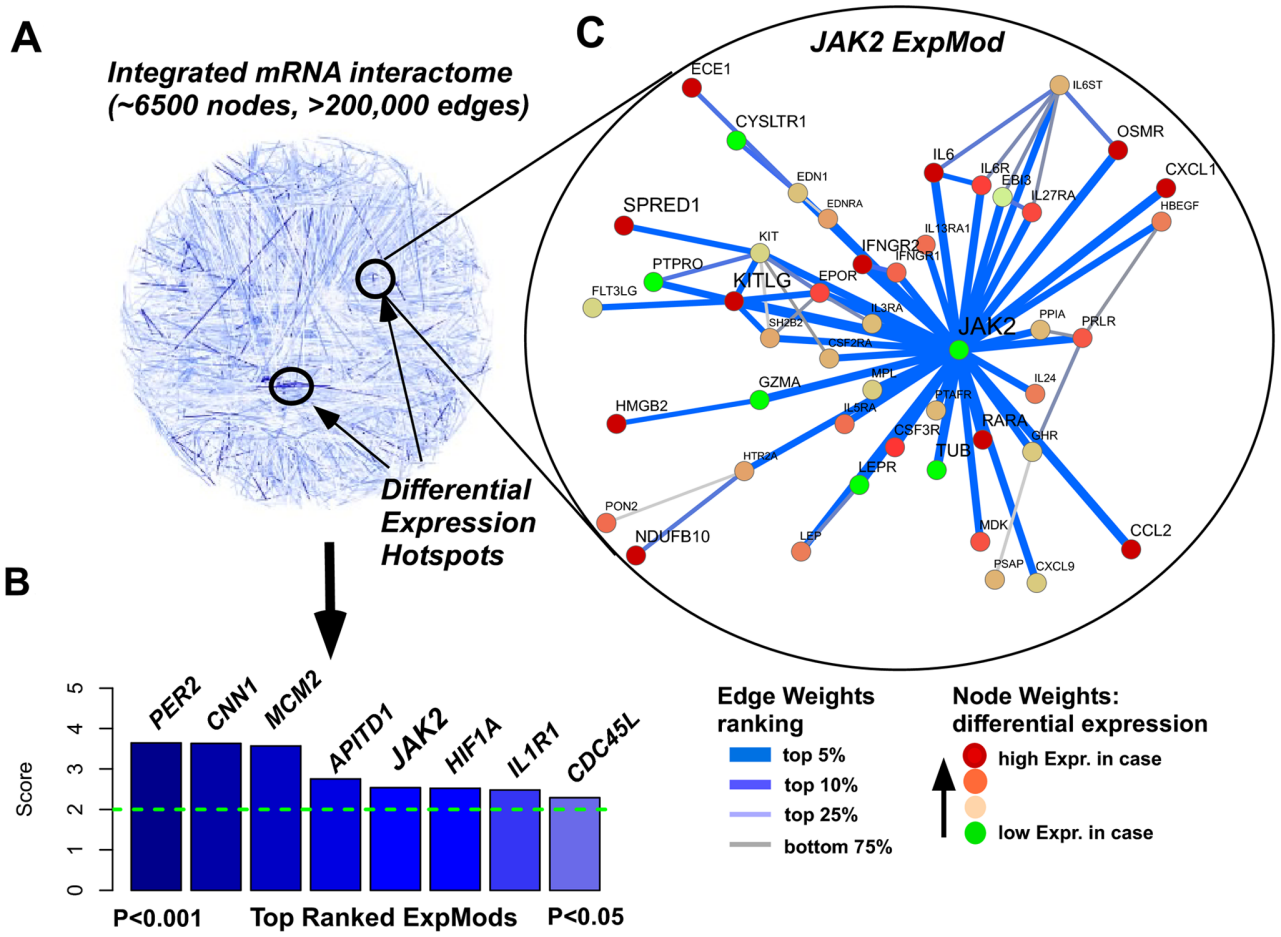
Top Ranked ExpMods and JAK2 ExpMod. Derivation of the JAK2 hotspot associated with aortic dissection: (A) The spin-glass module detection algorithm is applied to an integrated mRNA expression interactome to identify differential expression hotspots. The edges in the network are weighted according to the average of the absolute regularized t-statistics of the genes making up the edge (Methods). The edge weights are colour coded as indicated. (B) Detection of 8 non-redundant highly exclusive differential expression hotspots/modules (ExpMods) associated with aortic dissection, ranked according to their modularity values and all passing an adjusted *P*-value threshold of 0.05. The gene symbols of the seeds are shown. (C) Zoomed-in version of the JAK2 ExpMod. Both edge color and width encode the edge weights, as indicated. The Limma t-statistics of each gene (node) are shown as indicated. Observe how JAK2 defines the hub of this module, and forms dense subnetworks with other genes that are strongly differentially expressed e.g. IL-6/IL-6R, CCL2, KITLG and EPOR.

In order to obtain functional insights, we decided to apply a powerful functional supervised analysis method, which performs supervised feature selection at the level of protein interaction subnetworks. This approach can identify small yet subtle, coordinated changes in differential expression affecting genes that are part of the same module or pathway. Hence, we integrated the gene expression array data with a highly curated and comprehensive human protein interaction network, resulting in an integrated network of 6437 proteins and 258954 interactions (edges). We then used a module detection algorithm to identify “hotspots” of differential expression in the network (figure 3A). The algorithm first performs local greedy searches around specific seeds to identify modules, i.e. gene subnetworks of high modularity (edge density), located close to the seeds [14]. Not all seeds generate modules, since some seeds may represent isolated nodes of association. In fact, of the 100 seeds (the 100 top ranked genes from Limma analysis), only 55 generated modules, with 42 of these containing at least 10 genes. Statistical significance of the modularity values associated with these 42 modules was assessed further using a Monte-Carlo analysis whereby expression profiles were randomized across the network keeping the network topology fixed. We identified 8 gene modules all passing an adjusted P-value threshold of 0.05 (figure 3B). Thus, these 8 modules represent hotspots of differential expression associated with aortic dissection. We call these hotspots “Expression Modules (ExpMods)”. The 8 hotspots/ExpMods were derived from the following seed genes: PER2, CNN1, MCM2, APITD1, JAK2, HIF1A, IL1R1 and CDC45L, and were largely non-overlapping, representing fairly unique modules. GSEA performed on each ExpMod ascertained their biological significance. For example, IL1R1 module is enriched for TLR signaling, APITD1 module is enriched for DNA repair genes, and CDC45L module is enriched for complement and coagulation cascades. HIF1A module relevant to hypoxia also comes up.

JAK2 appeared both in the top 25 gene list from Limma analysis and in the 8 hotspots. The JAK2 module containing totally 112 genes is highly enriched for cytokines and receptors, e.g. IL-6, kit ligand (KITLG), Chemokine (C-C motif) ligand 2 (CCL2), Chemokine (C-X-C motif) ligand 1 (CXCL1), IL-24, IL-6R, IL-5RA, IL-27RA, IL-13RA1, EPOR, INFGR, CSFR, OSMR and PRLP. Interestingly, most of the cytokines and receptors are up-regulated, while JAK2 is strongly down-regulated in AAD patients. As shown in the zoomed-in version of the JAK2 ExpMod (figure 3C), JAK2 defines the hub of this module and forms dense subnetworks with other genes that are also differentially expressed.

### Protein-protein Interaction Involving JAK2 by STRING

**Figure 4:**
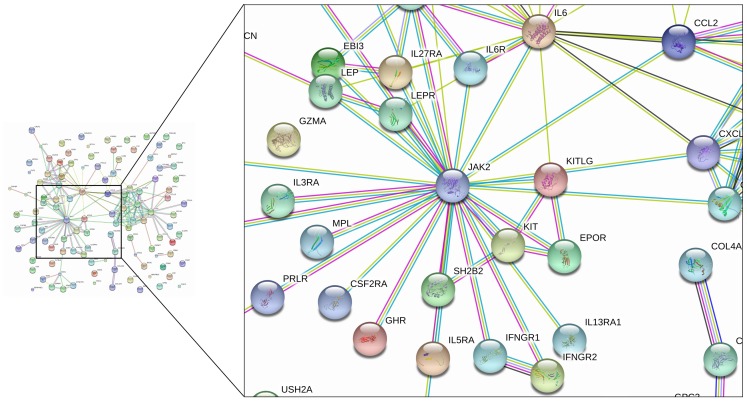
JAK2 protein-protein interaction network by STRING analysis. The network nodes are proteins. Seven differently colored lines represent 7 types of evidence used in predicting the associations. A red line indicates the presence of fusion evidence; a green line - neighborhood evidence; a blue line - coocurrence evidence; a purple line - experimental evidence; a yellow line - textmining evidence; a light blue line - database evidence; a black line - coexpression evidence.

As an additional verification of the JAK2 ExpMod detected by our integrative network algorithm, protein-protein interaction involving JAK2 was visualized using STRING database (figure 4). The image summarizes the network of predicted associations for a particular group of proteins. The edges represent the predicted functional associations based on 7 different types of evidence including fusion evidence, neighborhood evidence, coocurrence evidence, experimental evidence, textmining evidence, database evidence and coexpression evidence. Consistent with the structure of ExpMod, JAK2 acts as the center of the network, and has predicted functional associations with other objects such as IL-6/IL-6R, CCL2, KITLG and EPOR.

### Validation of the JAK2 Expression Module

**Figure 5:**
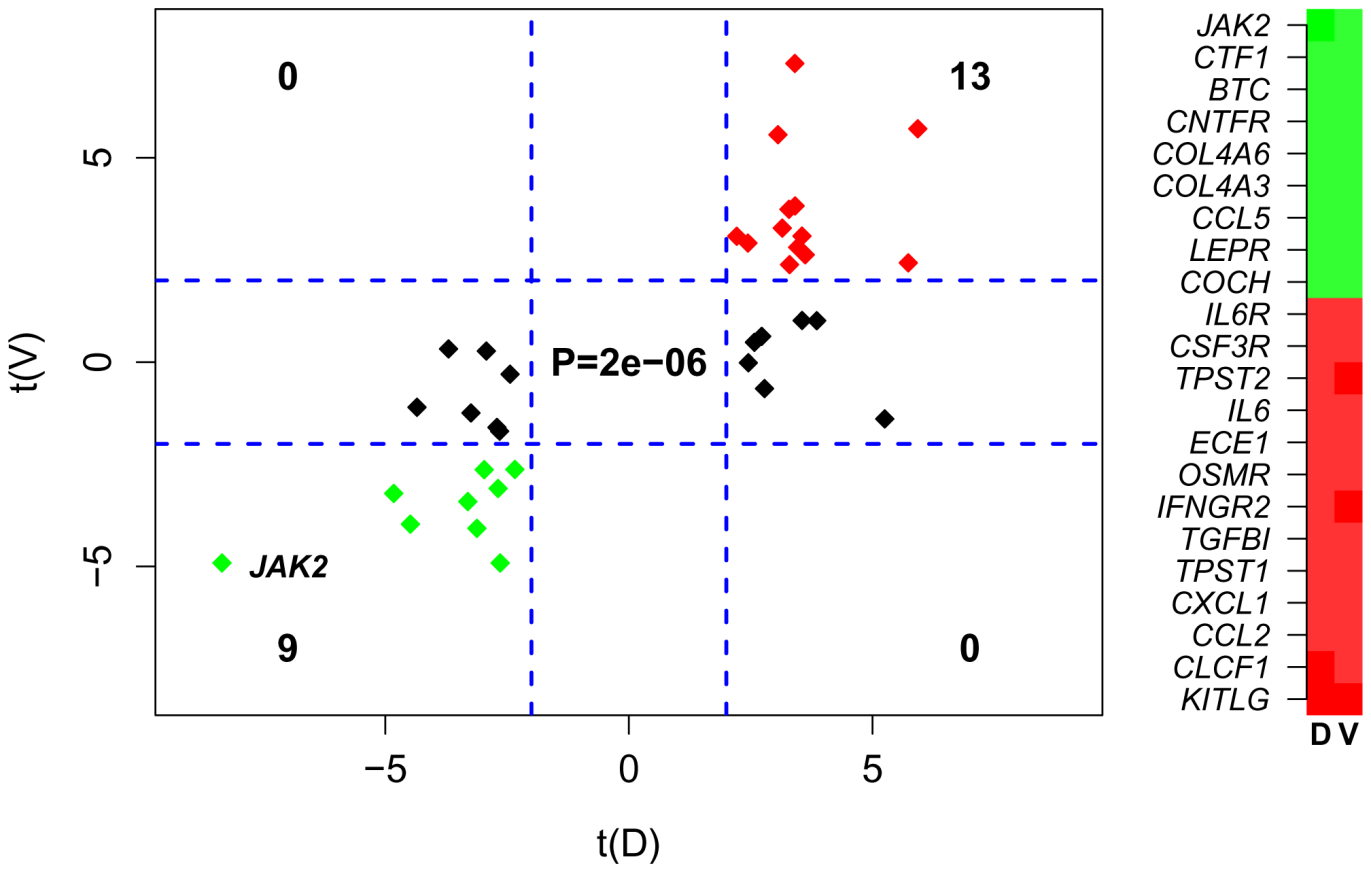
Validation of the JAK2 Expression Module. Left panel: scatterplot of regularised limma t-statistics of JAK2 Expression Module members in our discovery data set (x-axis, t(D)) against an independent validation set (Weis-Mueller et al 2006) (y-axis, t(V)). Positive statistics indicate overexpression in AAD cases relative to normal controls. Blue dashed lines indicate the lines of nominal significance P = 0.05. Fisher’s exact test P-value, reflecting agreement between two sets is given. Right panel: heatmap of the differential expression statistics in discovery and validation set for the 22 JAK2 module members showing consistency between the two sets. Green: underexpressed in AAD. Red: overexpressed.

To obtain further evidence for the significance of the expression changes seen in the inferred JAK2 module, we evaluated the JAK2 module in an independent gene expression data set of 4 AAD cases and 4 controls [15]. Validating the JAK2 module, we observed that many members of this module also showed significant differential expression changes between AAD and controls with the same directionality as in our discovery set (Fisher exact test P<1e-5, figure 5). In total we identified 22 genes (this included JAK2, IL6, IL6R) which showed the same pattern of differential expression, with no genes showing an opposite differential expression pattern (figure 5). Thus, the RT-PCR data, as well as the independent microarray data, which was generated on a different platform (Affymetrix), confirm the significance of the differential expression changes of JAK2 and many members of its interaction neighborhood.

## Discussion

The present study explored a new strategy, based on interaction networks, to investigate the molecular mechanisms underlying AAD.

Patients with heritable connective disorders, such as Marfan syndrome patients with a defect of the glycoprotein fibrillin-1, and Ehlers-Danlos syndrome patients with a type III-procollagen disorder are known to develop aortic dissection [17], [18]. But molecular features underlying aortic dissection without connective tissue diseases remain poorly understood. It was proposed that some stable dysfunctions in gene expression may underlie the development of this kind of aortic dissection as well.

The most common approach to genomic analysis starts with the identification of differentially expressed genes and subsequent biological interpretation using Gene Set Enrichment Analysis. According to our definition, Limma analysis of the microarray data identified 2737 differentially expressed genes between 7 aortic dissection cases and 5 controls. Of the top 25 differentially expressed genes, 17 genes were up-regulated and 8 were down-regulated in cases.

However, the common approach may miss important biological pathways, because the inference does not take the pathway or network structure into account and because changes affecting individual features are often of a small magnitude. Thus, a number of statistical approaches have emerged using the pathway/network structure in the inference procedure. Here we used a novel integrative approach to infer network modules, which subsequently facilitated the biological interpretation [14]. By this integrative network algorithm, interactome hotspots associated with aortic dissection were identified. Among them, we found a JAK2 module, which was validated in an independent gene expression microarray data set, enriched for cytokines and receptors, including IL-6, IL-6R, CCL2 and IFNGR2.

That many cytokines are implicated in AAD is well supported by previous literature: for instance, IL-6, INF gamma and CCL2, were observed to be significantly increased in AAD patients [19]. The previous microarray study also observed that IL-2, -6 and -8 genes were up-regulated in aortic dissection [15]. It has also been noted that IL-6 is secreted at high levels in human aortic aneurysm disease [20], [21]. These data indicate an inflammatory process characterized by abnormal expression of cytokines in aortic disease.

Vascular inflammation is a common pathologic and physiological response in diverse cardiovascular disease processes, including atherosclerosis, myocardial infarction and congestive heart failure [22]–[25]. Furthermore, IL-6 is found to be linked with the development of coronary disease and atherosclerosis [26], [27].

Taking all the factors together, it is reasonable to hypothesize that chronic inflammation may probably play a contributory role in the pathogenesis of aortic dissection. However, the precise relationship between inflammation and AAD remains complicated and the specific pattern of the inflammation as well as the key regulators is still pending.

Therefore, when JAK2, one of the top 25 genes from Limma analysis, came up also as the hotspot, it was of particular interest to further discuss its potential biological significance. The JAK family plays a critical role in growth, development, survival and differentiation through signal transduction of many cytokine receptors [28]. STAT3, a downstream transducer activated by JAKs, was found to be involved in vascular abnormalities. Frequent cerebral and coronary artery ectasias and aneurysms were observed in patients with STAT3 deficiency [29], [30], suggesting JAK/STAT-dependent signaling may be implicated in the maintenance of vessel integrity.

To predict the regulatory network involving JAK2 in AAD, we thus attempted to visualize the interaction among JAK2 and the differentially expressed cytokines and receptors. By using different bioinformatics databases, we pictured the JAK2 expression module and the protein-protein interaction network and got consistent results that JAK2 defines a hub and hence acts as the key regulator in the cluster.

JAK2 is known to be implicated in signaling by various receptor families. It binds to the receptors and keeps them at the cell surface in the absence of ligand, while in the presence of cytokine it starts the transduction through signaling pathways e.g. JAK/STAT. Upon cytokine binding, JAK2 also induces rapid receptor degradation. Therefore the homeostasis of JAK2 serves an important role in the cytokine sensitivity of cells [31]. As in the case of IL-5, JAK kinase activity was required for receptor down-regulation via degradation and internalization, and thereby less JAK2 might up-regulate IL-5R signaling capacity [32]. Unrestrained signaling resulting from receptors not desensitized sufficiently could potentially result in various inflammatory disorders such as hypereosinophilic syndrome [33]. Our current finding proposes that the significant reduced level of JAK2 in AAD may have an impact on the expression of cytokines and more considerably on the degradation and desensitization of cytokine receptors, result in over-expressed receptors and magnified extracellular signals, and thus play a key role in the inadequate inflammatory reaction in aortic wall, which consequently contributes to the occurrence of AAD.

The most common pathology associated with AAD is medial degeneration characterized by loss and fragmentation of elastic fibers and mucoid ground substance in the media, in some cases accompanied by smooth muscle cell loss. An immunohistochemical study showed that CD3^+^ cells with altered morphology and CD68^+^ cells were present in diseased ascending aortas and involved in media degeneration [34]. The JAK2 ExpMod is compatible with the potential existence of inflammatory cells in AAD, indicating an inadequate inflammation regulated by JAK2 associated with media degeneration.

In our patient set, histological investigation with HE stain revealed predominantly mucoid degeneration of elastic fibers. However, there was band of smooth muscle cell loss in one of the seven patients. It would be expected that a different cellular composition might influence the gene expression data. Further evaluation relying on gene expression signatures of the normal cell subtypes may help to alleviate the impact of cellular heterogeneity bioinformatically.

It is important to emphasize that the integrative network analysis may provide a new framework for understanding the molecular basis underlying AAD and is promising to illustrate the tangled relationship of differentially expressed genes in complicated diseases. The JAK2 ExpMod implicating the interaction between JAK2 and cytokines/receptors was only made possible through the integrative analysis strategy.

In summary, the present study described the differentially expressed gene profile of ascending aorta from patients with Stanford type A acute aortic dissection and predicted specific underlying biological processes involved in aortic dissection formation. The data demonstrated an inflammation with over-expressed cytokines and receptors in AAD. Furthermore, the integrative network analysis identified JAK2, as the key regulator of this inflammatory process. The results obtained may lead to further studies to explore the strategy for targeted intervention of vascular inflammation in AAD.

## Supporting Information

Figure S1 Histological assessment of the dissected aortas. (A) Medial degeneration associated with focal accumulation of basophilic ground substance. (B) Band of cellular loss in the aortic media. (HE stain, Magnification 200×).
(TIF)

Figure S2 The Bayesian regularized t-test analysis and False Discovery Rate (FDR) analysis. Left panel: histogram of P-values from the Bayesian regularized t-test analysis. Right panel: plot (red curve) of the number of expected false positives (E[nFP], y-axis) against number of positive tests (nP, x-axis). The ratio E[nF]/nP defines the q-value or False Discovery Rate (FDR). The dashed green line indicates the case of no association, where E[nF] = nP. The vertical dashed line indicates the point at which FDR = 0.05.
(TIF)

Table S1 The GSEA enrichment table for the upregulated genes.
(XLS)

Table S2 The GSEA enrichment table for the downregulated genes.
(XLS)
