# Supplementary figures and images for: JAK2-Centered Interactome Hotspot Identified by an Integrative Network Algorithm in Acute Stanford Type A Aortic Dissection
Source: PLoS One. 2014 Feb 24;9(2):e89406. doi: 10.1371/journal.pone.0089406 (PMC3933461; doi:10.1371/journal.pone.0089406)

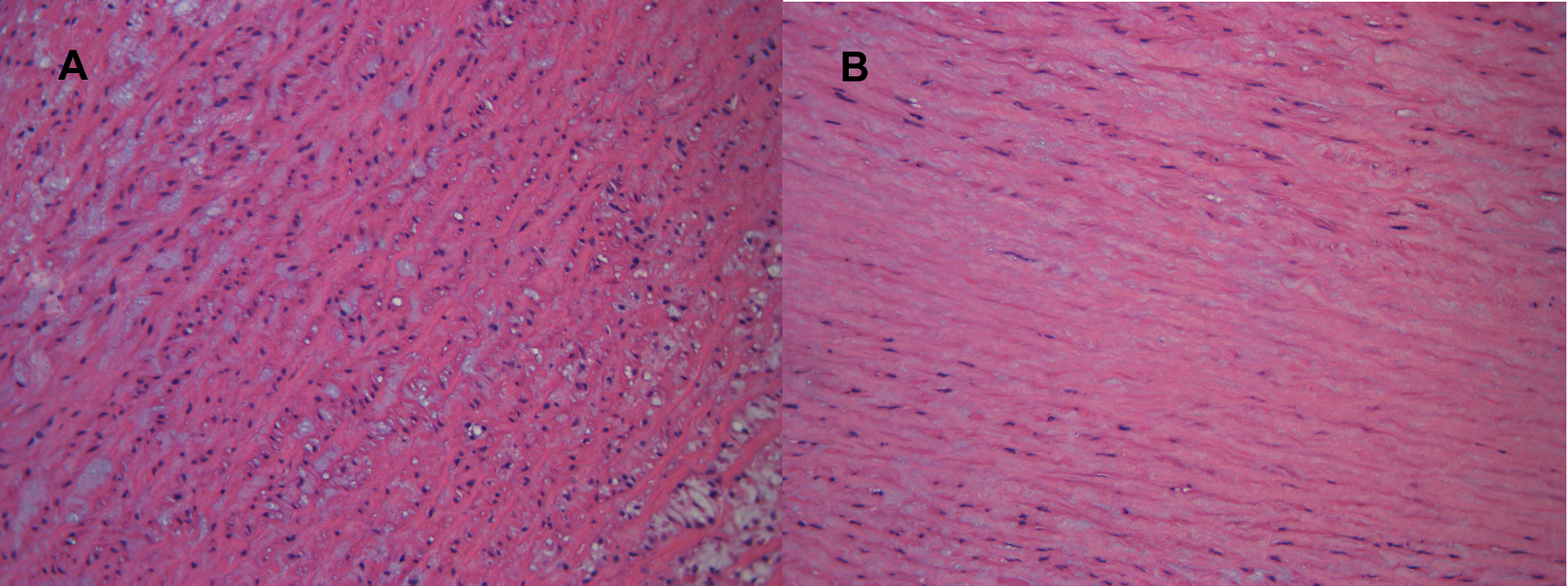

Supplement: Figure S1 — Histological assessment of the dissected aortas. (A) Medial degeneration associated with focal accumulation of basophilic ground substance. (B) Band of cellular loss in the aortic media. (HE stain, Magnification 200×). (TIF) [file pone.0089406.s001.tif]

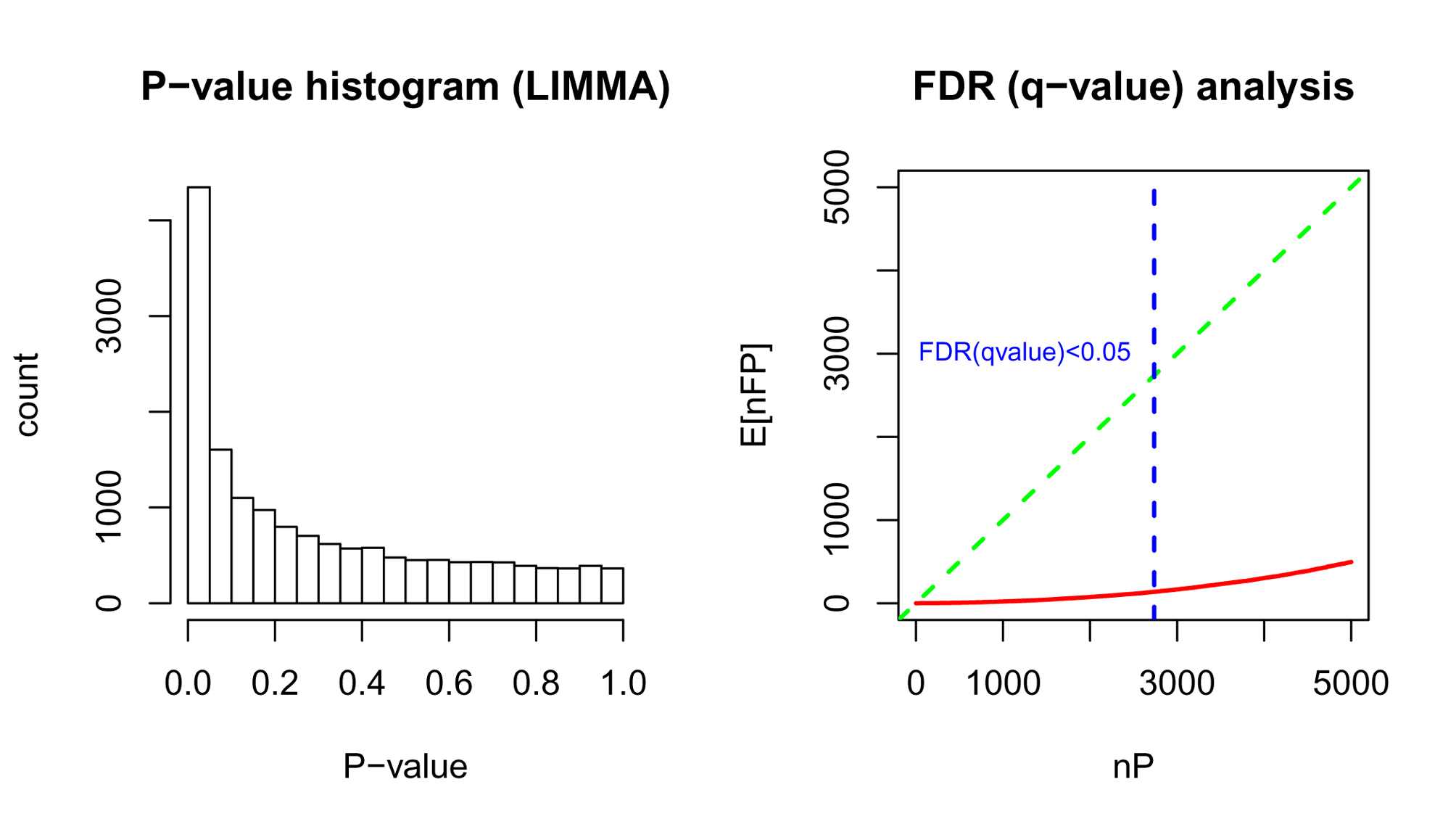

Supplement: Figure S2 — The Bayesian regularized t-test analysis and False Discovery Rate (FDR) analysis. Left panel: histogram of P-values from the Bayesian regularized t-test analysis. Right panel: plot (red curve) of the number of expected false positives (E[nFP], y-axis) against number of positive tests (nP, x-axis). The ratio E[nF]/nP defines the q-value or False Discovery Rate (FDR). The dashed green line indicates the case of no association, where E[nF] = nP. The vertical dashed line indicates the point at which FDR = 0.05. (TIF) [file pone.0089406.s002.tif]
